# COVID-19 Pandemic: Impact on Cancer Patients

**DOI:** 10.3390/ijerph191912470

**Published:** 2022-09-30

**Authors:** Monika Rucinska, Sergiusz Nawrocki

**Affiliations:** Department of Oncology, Collegium Medicum, University of Warmia and Mazury in Olsztyn, 10-228 Olsztyn, Poland

## 1. The COVID-19 Pandemic and Associated Changes in Medical Care

In December 2019, there were first reports of an atypical pneumonia detected in Wuhan city, China. The infectious agent was soon identified as a novel enveloped beta-coronavirus. In February 2020, the World Health Organization (WHO) designated the disease COVID-19 that is caused by a severe acute respiratory syndrome coronavirus 2 (SARS-CoV-2). This is the third health crises caused by a beta-coronavirus. Previous problems were caused by the severe acute respiratory syndrome coronavirus (SARS-CoV) and middle east respiratory syndrome coronavirus (MERS-CoV). A total of ca 10,000 cases of infections were caused by these viruses worldwide [1,2]. In March 2020, the WHO made the assessment that COVID-19 can be characterized as a pandemic. The COVID-19 pandemic has become the most severe global health challenge since the Spanish Flu one hundred years ago [3]. The novel coronavirus has reached all countries, with over 600 million confirmed cases and more than 6.5 million deaths [4]. Most governments have been implementing drastic measures to slow down the spread, such as travel restrictions, social distancing, and lockdowns. The rapidly expanding COVID-19 pandemic and restrictions affected all areas of daily life, including medical care. The World Health Organization declared COVID-19 as a public health emergency of international concern. Many governments have decided to allow the reallocation of resources in health care and take drastic measures to limit activities in other areas than just fighting the pandemic. Human and economic resources have been directed toward countering the pandemic and caring for COVID-19 patients, while many diagnostic and therapeutic services for other patients have been postponed or canceled. Some diagnostic and treatment departments, and even some hospitals were transformed into ones dealing with only COVID-19 patients. The COVID-19 pandemic has also caused staff shortages. Some medical personnel became infected, some were quarantined, and some were redirected to fight the pandemic. In the wake of severe social distancing and lockdown, outpatient clinics were largely moving to telemedicine for non-pandemic-related health services. Many people have chosen themselves not to report to clinics, hospitals or for testing for fear of coronavirus infection [5]. Others have been unable to go to doctor’s appointments or examinations/imaging tests for economic and social reasons—limitations on public transportation, lack of options for their own transportation, separation from loved ones and family and lack of support from others [5]. In general, the access to health care for patients diagnosed with different diseases, including cancer patients has begun to be limited.

## 2. Impacts of Postponed Cancer Screening, Diagnosis and Treatment

The COVID-19 pandemic had a significant effect on the diagnosis and treatment of cancer patients worldwide, especially at the beginning of the pandemic [6,7,8]. The impact of the pandemic resulting in a government-imposed lockdown, mandated social distancing, inefficiencies in health care organizations has essentially led to a near-total halt in cancer screening in the first months of the pandemic. Furthermore, some cancer societies and organizations (for example the American Society for Clinical Oncology) have recommended delaying screening tests, such as mammography and colonoscopy [9]. In addition, many patients chose not to seek medical care or take part in cancer screening programs during the pandemic due to various medical and non-medical reasons [10,11]. In part, this is due to the fact that generalized fear of infection and contact with healthcare and social anxiety was prevalent. It is estimated that there were delays in more than 22 million cancer screening tests in the US during the first year of the COVID-19 pandemic [12]. In Poland, restrictions introduced in the beginning of the COVID-19 pandemic had a significant negative effect on breast cancer and cervical cancer screening programs. In April–May 2020, 94% fewer mammograms and 88% fewer cytological examinations were performed in the provinces analyzed compared to the previous year [13]. The total number of screening mammograms was reduced by 27% in 2020 compared with 2019 and a decrease in screening coverage rates was observed (38.5% in 2019 and 34.5% in 2020) [13,14]. In Belgium, only 50% of cytology tests were performed in the first four months of 2020 compared to the same period in 2019 [15].

Postponed or cancelled patient appointments and screening exams will result in a decrease in new cancer diagnoses and a delay in treatments. Decreased new cancer diagnoses were documented for many cancers by about 40–50% in comparison to the previous years’ [5]. Due to the restrictions on access to health services, including visits to the family doctor, screening tests and visits to specialists, cancer patients are diagnosed at more advanced stage. A rise in advanced breast cancer cases during the COVID-19 pandemic was reported in some countries [16,17,18]. Analyses based on predictive models have shown that even short-term suspension of preventive screening can have negative effects. In Canada, it is projected that withholding breast cancer screening for 3 months will result in a 7% decrease in the number of diagnosed cases of breast cancer per year, with an increase in the number of cases of cancer detected in advanced stages [19]. In Italy, it has been estimated that a 6-month delay in breast cancer screening will result in an increase in the stage of the disease from T1 to T2 at diagnosis in half of all women diagnosed with future breast cancer [20]. A statistically significant increase in the number of stage III breast cancer cases was observed in Poland in 2021 [21]. In Italy, a 6.9% reduction in newly diagnosed lung cancer cases was observed in 2020 in comparison to 2019 [22]. Newly diagnosed lung cancer patients in 2020 were more likely to be diagnosed in stage IV of the disease (72% vs. 67%) [22]. It is well known that patients diagnosed at a more advanced cancer stage have poorer prognosis.

Additionally delays or even cancellations in screening and clinic appointments caused the delay of the start of treatment. The prolonged waiting time for cancer diagnosis and treatment is harmful. A longer waiting time for cancer diagnosis and treatment contribute to higher mortality and lower survival and correlates with a more advanced stage of disease among breast cancer patients, and head and neck cancers. It has been shown that the five-year overall survival of patients treated for breast cancer was lower by about 5–7% when waiting longer from the first symptoms (up to 3 months vs. 3–6 months) [23]. Modeling presented by Sud et al. [24] suggested a clinically significant impact on the number of lives and years of life lost for the COVID-19-related extension of the 2-week-wait cancer referral pathway in the UK. The National Cancer Institute predicted an additional 10,000 deaths over 10 years in the United States from breast and colorectal cancers due to reduced access to cancer diagnosis and treatment in the early months of the pandemic [25]. A large UK modeling study has estimated an increase in lung cancer mortality rate of 5% up to 5 years after diagnosis [26]. A forecast published in April 2020 predicted nearly 18,000 more cancer deaths in the UK over the next year due to declines in diagnosis and delays in starting cancer treatment [26,27].

An analysis supported by the National Institutes of Health (NIH) confirmed that during the COVID-19 pandemic there were problems with recruitment to clinical trials [28]. Cantini et al. [22] showed a decrease in the proportion of lung cancer patients in clinical trials (5% vs. 7%).

During the first year of the pandemic, there were noted drops in numbers of patients treated with radiotherapy by 6–36% in cancer centers in Europe and North America [29,30,31,32].

In addition, follow-up appointments or imaging studies have been delayed at many clinics around the world.

The pandemic itself and the disruption of access to medical care have become a source of stress and anguish, which can have a negative impact on mental health. Severe anxiety from fear of infection and a decrease in social support from family members worsened the mental condition and well-being of cancer patients. Social distancing during a pandemic contributes to mental health disorders, loss of motivation and loss of self-worth [33]. Uncertainty of treatment plans and outcomes in a difficult pandemic situation can become a cause of additional suffering and stress for cancer patients and families. There were observably higher rates of psychologic distress among patients during the COVID-19 pandemic compared to non-pandemic periods. This includes distress from the pandemic in general, their cancer diagnosis, as well as distress about receiving adequate treatment [34]. Romito et al. [35] showed that 36% of patients with lymphoma under chemotherapy during the first phase of lockdown in Italy had anxiety, 31% of patients had depression and 36% of patients had post-traumatic stress disorder.

The COVID-19 pandemic has affected the quality of life and well-being of cancer patients, as well as survivors in follow-up. Ng et al. [36] found that cancer survivors were more likely than healthy individuals to report depressive symptoms and catastrophizing about the COVID-19 pandemic.

## 3. COVID-19 in Cancer Patients

Cancer patients are a population particularly vulnerable to the SARS-CoV-2 infection. Lee et al. in a large community-based survey showed that compared with individuals without cancer, cancer patients had a 60% increased risk of a positive COVID-19 test [37]. Current anticancer treatments (chemotherapy or immunotherapy) were linked to a 2.2-fold increased risk of a positive test. Cancer patients may be more prone to getting infected due to frequent contact with the health service and other patients, because they need to regularly visit hospitals and outpatient clinics to receive their therapy or control. The susceptibility of cancer patients to the SARS-CoV-2 infection may also be related to anticancer treatment and its complications.

Cancer patients develop more severe COVID-19 symptoms, which is possibly due to the systemic immunosuppressive state caused directly by tumor and indirectly by effects of anticancer treatment [38,39]. Cancer patients had been identified as a population with an increased risk of severe clinical events associated with infections—admission to the intensive care unit and need for invasive ventilation—in comparison to non-cancer individuals (39% vs. 8%) [1]. Patients with hematological malignancies had an even greater risk of severe symptoms associated with COVID-19 (66.7%) [40]. Kuderer NM et al. in a retrospective cohort study with 1035 COVID-19-positive patients with cancer reported that mortality in COVID-19 cancer patients were significantly higher than the general population [41]. In addition, the meta-analysis of 22 studies that evaluated the prevalence of cancer among patients infected with COVID-19, showed that cancer patients had a higher risk of severe/critical disease (OR = 3.91, 95% CI: 2.70–5.67) and mortality (odds ratio [OR] = 3.23, 95% CI: 1.71–6.13) than non-cancer patients [39]. However, a prospective cohort study by Lee et al. demonstrated that COVID-19 mortality in cancer patients was principally related to their advancing age and the presence of other severe comorbidities [42]. Additionally, it is known that anticancer systemic treatments (both chemotherapy and immunotherapy) are related to severe clinical events due to the SARS-CoV-2 infection [43,44]. There is no evidence that radiotherapy increases the risk of COVID-19 symptoms and complications of infection [40]. There is also a study reporting that some cancer patients did not appear to have a higher risk of severe COVID-19 than the general population—for example breast cancer patients [45]. Most of above studies are cohort studies and thus have some limitations.

There is also a suspicion that cancer survivors may have more severe COVID-19 course to patients without a history of cancer [30,38,43].

## 4. The Challenges of Post-Pandemic Cancer Care

Undoubtedly, the COVID-19 pandemic has had a huge impact on cancer diagnosis, treatment and prognosis. In particular, early detection of new cancer cases, diagnosis of people with suspected cancer and treatment of cancer during the COVID-19 pandemic were the challenges for the oncological health system. The pandemic caused delays in the start of treatment for patients with newly diagnosed malignancies and may have also caused interruptions in treatment. These situations could result in adverse oncologic outcomes. It is obvious that the long-term impacts of delayed cancer diagnosis and treatment, as well as delayed follow-up appointments during the COVID-19 pandemic will not be evident for some time [5]. Many institutions of care systems have implemented marketing campaigns to encourage people to re-enter the healthcare system for cancer screening [46]. It is difficult to predict how women who did not come in for a screening mammogram or cytology in the first months of the pandemic will behave—whether they will come in at a later date, after some time, or whether they may abandon the examinations altogether for an extended period of time.

As the COVID-19 pandemic took the health system by surprise, unfolding in unpredictable ways, it is important to assess how the pandemic has affected various aspects of cancer care so that its long-term effects in this patient population can be understood [5]. Although, the demands of cancer care are likely to increase in the future, driving up healthcare costs. Oncological centers should be prepared for potential further events related to this unpredictable pandemic. The National Comprehensive Cancer Network (NCCN) made recommendations for promoting mental well-being during the COVID-19 pandemic in cancer patients such as promoting physical fitness activities even at home, contact with nature, keeping in touch with family and friends and doing things one enjoys [47].
